# Low-Altitude Target Localization Method Based on Exogenous Radar with Multi-Base Station and 5G SSB Signals

**DOI:** 10.3390/s26072183

**Published:** 2026-04-01

**Authors:** Yike Xu, Gangyi Tu, Luyan Zhang, Yi Zhou, Meiling Xiong, Yang Li

**Affiliations:** 1 School of Electronic and Information Engineering, Nanjing University of Information Science & Technology, Nanjing 210044, China; 202383270294@nuist.edu.cn (Y.X.); 202383270189@nuist.edu.cn (L.Z.); 202412180619@nuist.edu.cn (M.X.); 202483270551@nuist.edu.cn (Y.L.); 2 School of Automation Engineering, Nanjing University of Information Science & Technology, Nanjing 210044, China; 202313360061@nuist.edu.cn

**Keywords:** 5G system, multi-beam, SSB detection and identification, RSRP

## Abstract

In this work, we propose a localization method based on an exogenous radar with multi-base station and the synchronization signal block (SSB) in 5G downlink signals. We combine physical cell identities (PCIs)-based identification with the extensive cancellation algorithm (ECA) to reconstruct and cancel the present strongest SSB signal, thereby obtaining reference signal receiving power (RSRP) values of them in descending order of strength. Then, we designed a two-stage localization method. Firstly, we determined the target’s coarse location based on the directional characteristics of different SSB beams. Subsequently, we compared the RSRP values extracted from the actually received signals against those pre-obtained when the target is at various reference points. The reference point corresponding to the closest match was selected as the estimated target position. We conducted simulations under various signal-to-noise ratio (SNR) levels, reference point densities, and signal jitter conditions. The simulation results demonstrate that the method outperforms techniques such as Fang’s method for time difference of arrival (Fang-TDOA) and observed time difference of arrival (OTDOA).

## 1. Introduction

In recent years, unmanned aerial vehicle (UAV) technology has become increasingly mature and gained widespread attention [1]. 5G technology, with its broad operating bandwidth, low transmission delay, robust communication capability, and high positioning accuracy, offers excellent performance in detecting low-altitude targets, such as UAVs.

Nowadays, increasing attention has been paid to low-altitude target localization, particularly for UAV detection [2,3,4]. Studies on radio frequency (RF)-based localization for aerial platforms highlight the growing importance of wireless sensing technologies in the field of low-altitude target localization. Among these approaches, 5G New Radio (NR) signals have been widely exploited due to their rich signal structures. For instance, the 5G SS-RSRP has been used in real-time distance estimation [5]. Additionally, the multi-beam features of 5G NR have been used to improve localization accuracy. Superimposed multi-beam RSRP and reference signal reception quality (RSRQ) have been used to assist deep learning for indoor localization [6]. Furthermore, the IMPos indoor mobile positioning system achieves high-precision positioning by using the RSRP of 5G multi-beam downlink signals [7].

Besides direct signal measurements, fingerprint localization has also been used in different ways [8,9,10]. In addition, hybrid localization methods have been proposed. A base station selection method based on geometric dilution of precision (GDOP) enhanced round-trip time (RTT), time difference of arrival (TDOA), and direction of arrival (DOA) in hybrid localization [11], whereas integrated 5G observations (TDOA, RTT, and angle) combined with the 5G and precise point positioning (PPP) method demonstrated improved performance of localization [12].

Beyond traditional communication-based localization methods, integrated sensing and communications (ISAC) has also attracted considerable attention in recent years for future wireless networks [13,14,15]. A multi-beam system for joint sensing and communication based on 5G NR realized high-precision estimation of distance, velocity, and direction of arrival for multiple targets [16]. A multi-base station approach, which fuses information from different resource bands and distributed nodes, was used to estimate the angles-of-arrival (AoAs) of multiple targets [17]. In this context, passive radar that utilizes communication signals such as 5G has become more and more important due to its ability to achieve target detection and localization without the need for cooperative transmitters. Existing research has explored the use of 5G signals as a source of illumination in passive radar systems for target detection [18] and imaging [19].

However, none of the methods above have addressed the method of target localization based on exogenous radar (a passive radar using signals of opportunity) with multi-base station and 5G SSB signals.

In this work, we propose a novel target localization method that utilizes SSB signals and their corresponding RSRP values from multiple 5G base stations, from the perspective of communication signal identification. By utilizing the multi-beam characteristics of the existing signals from 5G base stations and the 5G NR network, we can localize the target within a certain range. By constructing a comprehensive RSRP database and comparing real-time RSRP values of each signal against it, we can locate the target to the nearest reference position, which greatly reduces the localization error.

## 2. Materials and Methods

### 2.1. SSB Beam Identification

The SSB is mainly used for cell search and initial time–frequency synchronization in wireless communication networks. The study presented in [20] innovatively utilizes 5G SSB signals as an irradiation source for passive radar to detect moving targets, such as vehicles. According to 3GPP TS 38.211 [21], SSB consists of four orthogonal frequency division multiplexing (OFDM) symbols in the time domain and 20 resource blocks (RBs) in the frequency domain. At the physical layer, each SSB consists of four key components: primary synchronization signal (PSS), secondary synchronization signal (SSS), physical broadcast channel (PBCH), and demodulation reference signal (DM-RS).

#### 2.1.1. Single SSB Beam Identification and Cell Search

Given that PSS and SSS have a limited length and are chosen from finite candidate sequences, single SSB beam identification and cell search can typically be divided into three steps: coarse PSS synchronization, fine PSS synchronization, and SSS synchronization. Assume $SSB_{\max}$ represents the SSB with maximum received power. Since the positions of PSS and SSS are always fixed in a 5G system, the start of $SSB_{\max}$ can be estimated by detecting the PSS and SSS.

For PSS sequence detection, we correlate the received signal samples y(n) with all the candidate sequences of PSS $\left\{s_{PSS}(n)\right\}$. Assuming the symbol length of the PSS is $N_{p}$, the correlation results $\Phi_{PSS}^{m_{2}}(n)$ can be expressed as:

(1) $$\Phi_{PSS}^{m_{2}}(n)=\sum_{k=0}^{N_{p}-1}s_{PSS}^{m_{2}}(n)\cdot y^{\text{*}}(n+k)\;,\;\;m_{2}\in \{0,1,2\}$$

where $m_{2}$ is the index of the candidate PSS sequence, and k is the correlation lag.

Then, the starting position ${\hat{\varepsilon }}_{\max}$ of $SSB_{\max}$ and the detected PSS sequence number ${\hat{m}}_{2}$ can be obtained by finding the correlation peaks:

(2) $$\left\{{\hat{\varepsilon }}_{\max},{\hat{m}}_{2}\}=\arg\underset{n,m_{2}}{\max}\Phi_{PSS}^{m_{2}}(n\right)$$

Similarly, for SSS detection:

(3) $$\Phi_{SSS}^{m_{1}}(n)=\sum_{k=0}^{N_{s}-1}s_{SSS}^{m_{1}}(n)\cdot y^{\text{*}}(n+k)\;,\;\;m_{1}\in \{0,1,\ldots ,335\}$$

(4) $$\left\{{\hat{\varepsilon }}_{\max},{\hat{m}}_{1}\}=\arg\underset{n,m_{1}}{\max}\Phi_{SSS}^{m_{1}}(n\right)$$

Based on the PSS sequence number ${\hat{m}}_{2}$ and the SSS sequence number ${\hat{m}}_{1}$, the PCI number $N_{ID}^{cell}$ corresponding to the $SSB_{\max}$ can be calculated:

(5) $$N_{ID}^{cell}=3\times {\hat{m}}_{1}+{\hat{m}}_{2}$$

#### 2.1.2. Multi-Beam Extraction

Multi-beam extraction of 5G NR signals provides richer spatial information required for low-altitude target localization. The process involves the following steps:

- Identifying the peak SSB and its PCI

Coarse time synchronization is achieved via PSS/SSS detection, as detailed in Section 2.1.1.

- Extracting the DM-RS from the peak SSB

The coarse time synchronization in Section 2.1.1 yields the coarse starting point of $SSB_{\max}$ in the time domain. After synchronization, OFDM demodulation, and the fast fourier transform (FFT), we obtain the frequency-domain signal. Then, the DM-RS sequence can be extracted from signal subcarriers with the starting subcarrier index γ decided by $N_{ID}^{cell}$:

(6) $$\gamma =N_{ID}^{cell}-\left\lfloor N_{ID}^{cell}/4\right\rfloor \times 4$$

where “⌊ ⌋” denotes rounding down.

- Identifying SSB index

Depending on the subcarrier spacing and frequency range, we denote the maximum number of SSB series as $L_{\max}$. The possible SSB indices in the SS burst cluster are $bn\in \{0,1,2,\ldots ,L_{\max}-1\}$, and the index of $SSB_{\max}$ is $bn_{\max}$. According to TS 38.211 [21], the local DM-RS sequence is determined by $N_{ID}^{cell}$ and bn. To obtain the value of $bn_{\max}$, we correlate the demodulated DM-RS with all possible reference DM-RSs:

(7) $$R_{DM-RS}(bn)=\sum_{p_{s_{1}}\in P_{s_{1}}}\left\|c_{bn}(p_{s_{1}})\cdot d_{bn_{\max}}^{\text{*}}(p_{s_{1}})\right\|$$

where $P_{s_{1}}$ is the set of all subcarrier positions occupied by the DM-RS signal in the time–frequency resource grid. $p_{s_{1}}$ is the indices of the subcarriers of all the DM-RS in $P_{s_{1}}$. $c_{bn}(p_{s_{1}})$ is the local DM-RS sequence in the bnth SSB, $d_{bn_{\max}}(p_{s_{1}})$ is the DM-RS sequence in $SSB_{\max}$, and “‖ ‖” denotes the modulo operation.

Then the index $bn_{\max}$ for $SSB_{\max}$ is:

(8) $$bn_{\max}=\arg\underset{bn}{\max}R_{DM-RS}(bn)$$

- Multi-beam extraction

According to 3GPP TS 38.211 [21], SSB spacing has two modes $\Delta l_{1}$ and $\Delta l_{2}$, forming the spacing matrix α. Assume $\alpha =\left[\Delta l_{1},\Delta l_{2},\Delta l_{1},\Delta l_{2},\ldots ,\Delta l_{1},\Delta l_{2},\ldots \right]\in {\mathbb{R}}^{1\times (L_{\max}-1)}$ represents the SSB interval vector. Thus, for each SSB in the synchronization signal (SS) burst set, the initial start sample ${\hat{\varepsilon }}_{bn}$ can be calculated as:

(9) $${\hat{\varepsilon }}_{bn}=\left\{\begin{array}{c} {\hat{\varepsilon }}_{\max}+\sum_{l=bn}^{bn_{\max}-1}\alpha (l)\;,\;bn<bn_{\max}\\ {\hat{\varepsilon }}_{\max}+\sum_{l=bn_{\max}}^{bn-1}\alpha (l)\;,\;bn>bn_{\max} \end{array}\right.$$

Subsequently, we fine-tune and update the initial position of each SSB signal to get the precise start samples for multi-beam extraction.

#### 2.1.3. Aliased SSB Beam Identification

In outdoor scenarios, the received signals generally consists of target echoes, direct-path signals and multipath noise caused by surrounding structures [22]. Conventional signal processing commonly selects the SSB with the highest power for physical cell information extraction, potentially misidentifying the PCI of the target echo and causing localization errors. After performing frequency offset estimation and frame timing synchronization on the aliased signals, we adopt the ECA [23] to overcome this limitation and sequentially detect all the SSBs, enabling the sequential detection of different cells.

We employ a high-precision frequency offset estimation method utilizing both PSS and SSS [23]. After acquiring the position of $SSB_{\max}$ through timing synchronization, the PSS sequence $s_{PSS}(n)$ and the SSS sequence $s_{SSS}(n)$ can be extracted from the received signals $y({\hat{\theta }}_{PSS}+n)$ and $y({\hat{\theta }}_{SSS}+n)$:

(10) $$y({\hat{\theta }}_{PSS}+n)=s_{PSS}(n)\cdot \exp(j2\pi \frac{{\hat{\theta }}_{PSS}+n}{N}\varepsilon )$$

(11) $$y({\hat{\theta }}_{SSS}+n)=s_{SSS}(n)\cdot \exp(j2\pi \frac{{\hat{\theta }}_{SSS}+n}{N}\varepsilon )$$

where ${\hat{\theta }}_{PSS}$ and ${\hat{\theta }}_{SSS}$ are the timing offsets of the PSS and SSS, ε is the fractional frequency offset, and N is the number of FFT points.

Then, the time domain of the received signals can be computed as:

(12) $$r_{PSS}(n)=y({\hat{\theta }}_{PSS}+n)\cdot s_{PSS}^{\text{*}}(n)={\left|s_{PSS}(n)\right|}^{2}\cdot \exp(j2\pi \frac{{\hat{\theta }}_{PSS}+n}{N}\varepsilon )$$

(13) $$r_{SSS}(n)=y({\hat{\theta }}_{SSS}+n)\cdot s_{SSS}^{\text{*}}(n)={\left|s_{SSS}(n)\right|}^{2}\cdot \exp(j2\pi \frac{{\hat{\theta }}_{SSS}+n}{N}\varepsilon )$$

Next, we perform relevant operations on $r_{PSS}(n)$ and $r_{SSS}(n)$, and denote the result as λ:

(14) $$\lambda =\sum_{n=0}^{N-1}r_{PSS}^{\text{*}}(n)\cdot r_{SSS}(n)=\exp(j2\pi \frac{{\hat{\theta }}_{SSS}-{\hat{\theta }}_{PSS}}{N}\varepsilon )\cdot \sum_{n=0}^{N-1}{\left|s_{PSS}(n)\right|}^{2}\cdot {\left|s_{SSS}(n)\right|}^{2}$$

where $N_{CP}^{short}$ is the length of the short cyclic prefix. Since the positions of the PSS and SSS signals within the SSB sequences are fixed, the timing deviation between them is constant:

(15) $${\hat{\theta }}_{SSS}-{\hat{\theta }}_{PSS}=2N+2N_{CP}^{short}$$

Therefore, Equation (14) can also be expressed as:

(16) $$\lambda =\exp(j2\pi \frac{2N+2N_{CP}^{short}}{N}\varepsilon )\cdot \sum_{n=0}^{N-1}{\left|s_{PSS}(n)\right|}^{2}\cdot {\left|s_{SSS}(n)\right|}^{2}$$

The fractional frequency offset estimate can be calculated using Equation (17) defined by the fractional frequency offset:

(17) $$\hat{\varepsilon }=\frac{N}{4\pi (N+N_{CP}^{short})}\cdot ∠\lambda$$

Then, we perform frequency offset correction on the received signal y(n):

(18) $$y^{\prime }(n)=y(n)\cdot \exp(-j2\pi \hat{\varepsilon }n/N)\;,\;n=0,1,\ldots ,N-1$$

**Figure 1:**
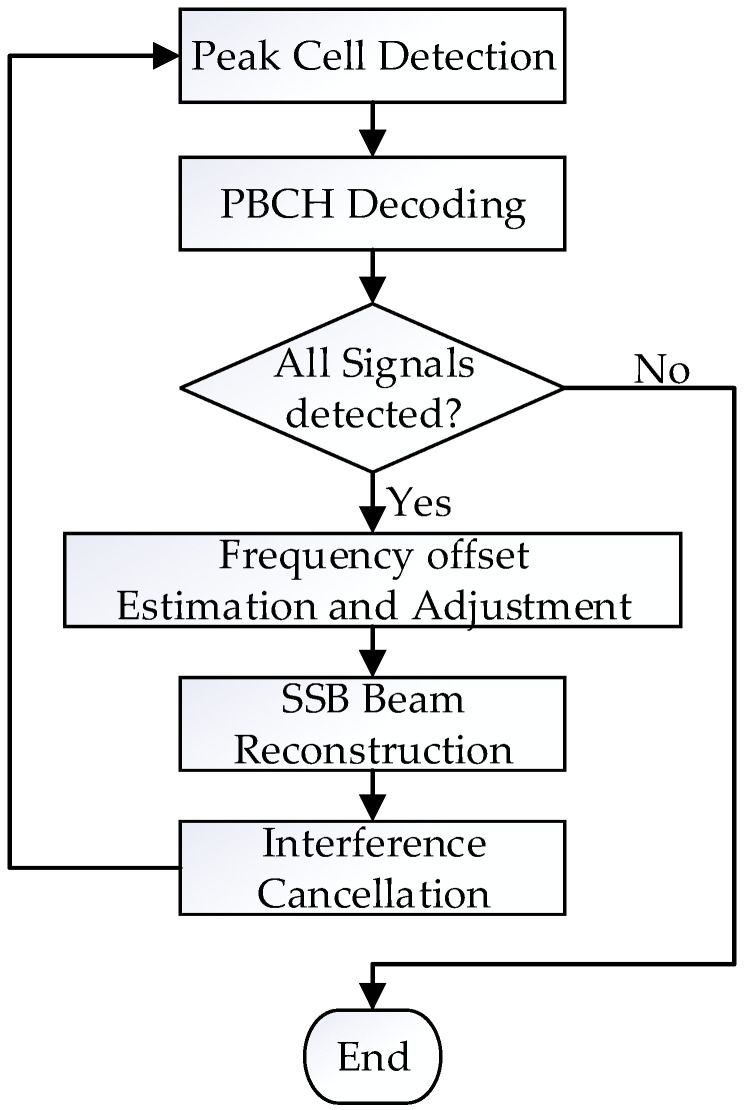
The structure of the ECA.

Upon accurately correcting frequency offset and achieving SSB beam timing synchronization, we use the ECA described in Figure 1 to reconstruct and cancel the signals.

The detailed progress is shown as follows:

- **Peak cell detection:** Since the peak cell possesses the highest power, it exhibits maximum correlation with the corresponding candidate sequences of PSS and SSS, thereby yielding the PCI of the peak cell.
- **PBCH decoding:** Within the SSB structure, the positions of various components are fixed. Using the identified PCI, the corresponding DM-RS and the data part of PBCH can be extracted from the SSB beam. Then we use the eight candidate DM-RS sequences to perform channel estimation to determine the index of the detected SSB beam. Finally, we perform channel equalization on the data portion and decode it to obtain the PBCH payload.
- **CRC check:** The PBCH channel in the 5G system contains 24-bit CRC check information which is used to determine whether the signal has been successfully detected and decoded. If the CRC check succeeds, we proceed to the subsequent steps; if the CRC check fails, we terminate the process.
- **SSB Beam Reconstruction:** After frequency offset estimation and correction on the received signal utilizing both PSS and SSS, we reconstruct the PSS, SSB, DM-RS, and PBCH based on the decoded PCI and MIB information in the sequence and map them onto the SSB beam currently being detected. Finally, we modulate them into the time-domain signal *y_SSB_*(*n*) via IFFT.
- **Interference Cancellation:** The original SSB signal and its delayed versions collectively form a subspace matrix. Using the least squares method, the received signal is projected onto this subspace for interference cancellation, thereby attenuating the peak cell signal and its multipath signals. The detailed steps of interference cancellation are as follows:

Let L be the length of the received signal and K be the maximum delay in the received signal. Then, $y^{\prime }(n)$ can be expressed as:

(19) $$y^{\prime }(n)={\left[y^{\prime }(0),y^{\prime }(1),\ldots ,y^{\prime }(L-1)\right]}^{T}$$

The L×K-order reference matrix V formed by the reconstructed SSB signal and its delayed versions can be expressed as:

(20) $$V=\left[\begin{array}{cccc} y_{SSB}(1) & 0 & \ldots & 0\\ y_{SSB}(2) & y_{SSB}(1) & \ldots & 0\\ \ldots & \ldots & \ldots & \ldots \\ y_{SSB}(L) & y_{SSB}(L-1) & \ldots & y_{SSB}(L-K+1) \end{array}\right]$$

Based on the least squares criterion:

(21) $$W=\arg\underset{w}{\min}\left\|y^{\prime }(n)-VW\right\|$$

where W denotes the subspace coefficients:

(22) $$W={\left[w_{1},w_{2},\ldots ,w_{K}\right]}^{T}$$

Let the derivative of Equation (21) to zero, then we can have:

(23) $$\frac{\partial ({\left\|y^{\prime }(n)-VW\right\|}_{2}^{2})}{\partial W}=0$$

We project the received signal onto the subspace using the least squares method for interference cancellation, so as to eliminate the impact of the peak cell signal and its multipath components. From Equation (23), we can have:

(24) $$W={\left(V^{H}V\right)}^{-1}V^{H}y^{\prime }(n)$$

where H denotes the conjugate transpose.

The remaining signal after interference cancellation can be defined as:

(25) $$y_{new}(n)=y^{\prime }(n)-VW=y^{\prime }(n)-V{\left(V^{H}V\right)}^{-1}V^{H}y^{\prime }(n)$$

- **Restart from peak cell detection and repeat the beam reconstruction and interference cancellation process in** *y_new_*(*n*) **until the CRC check fails:** We use the CRC check to determine whether the signal has been successfully decoded or not. Through an iterative structure, different cells will be detected sequentially in descending order of power until all detectable cells are identified.

### 2.2. Identification and Calculation of RSRP

RSRP refers to the strength of the signals measured by terminals in LTE/5G networks. In a 5G NR system, RSRP measurements are usually obtained based on SSS and DM-RS. It has been widely adopted as a key metric in various localization and predictive modeling studies, including deep-learning-based indoor localization using the RSRP and reference signal reception quality (RSRQ) of superimposed multi-beam signals [6], LSTM-based prediction of future time slot RSRP for UAV movement adjustment [24], simultaneous estimation of signal propagation models and base station locations [25], and outdoor environment applications integrating RSRP with deep learning techniques [26,27,28].

To calculate the RSRP value for reference signals, we first calculate the average received power of the SSS signal:

(26) $$P_{SSS}(bn)=\frac{1}{N_{s}}\sum_{p_{s_{2}}\in P_{s_{2}}}s_{bn}(p_{s_{2}})\cdot s_{bn}^{\text{*}}(p_{s_{2}})$$

where $P_{s_{2}}$ is the set of all subcarrier positions occupied by the SSS signal in the time–frequency resource grid, $p_{s_{2}}\in P_{s_{2}}$ denotes the indices of the subcarriers in $P_{s_{2}}$ and $s_{bn}(p_{s_{2}})$ denotes the SSS in the SSB sequence at subcarrier index bn.

Similarly, the average received power of the DM-RS signal can be calculated as follows:

(27) $$P_{DM-RS}(bn)=\frac{1}{N_{d}}\sum_{p_{s_{1}}\in P_{s_{1}}}d_{bn}(p_{s_{1}})\cdot d_{bn}^{\text{*}}(p_{s_{1}})$$

where $N_{d}$ is the length of the DM-RS sequence. Then, the SS-RSRP value $P_{bn}$ can be calculated as:

(28) $$P_{bn}=\frac{P_{SSS}(bn)\cdot N_{s}+P_{DM-RS}(bn)\cdot N_{d}}{N_{s}+N_{d}}$$

In this work, signals propagating over different paths experience varying attenuation determined by their transmission distances, leading to different RSRP values. The RSRP value of each signal, reconstructed using the ECA [23], is matched against the pre-constructed database of RSRP measured at different reference locations. The target position is then determined as the reference point exhibiting the closest RSRP match, thereby significantly enhancing positioning accuracy.

### 2.3. Multi-Base Station Overlapping Signal Model and Specific Positioning Method

**Figure 2:**
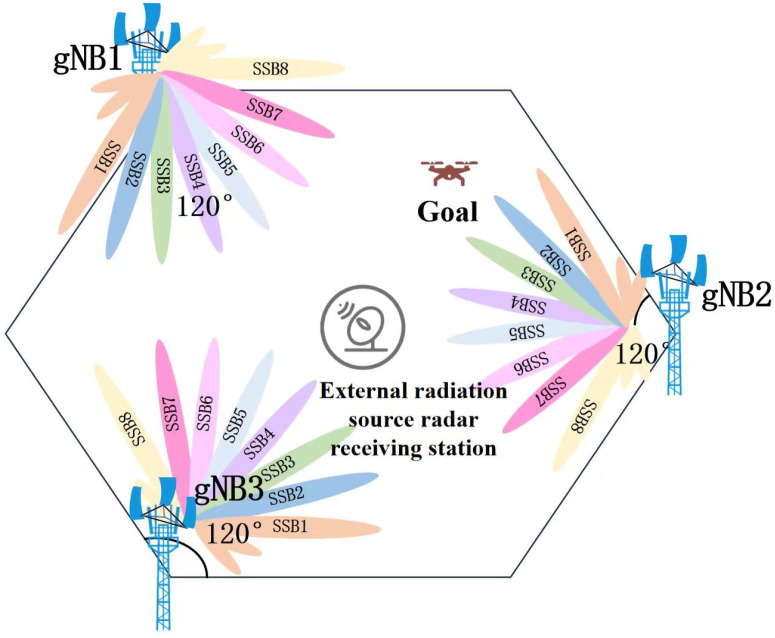
Signal aliasing system model in 5G networks.

This paper innovatively proposes an RSRP-assisted localization method with overlapping SSB signals from multi-base station. Figure 2 illustrates a simulated system model of overlapping signals from three 5G base stations. A regular hexagonal structure is employed, wherein each base station covers a 120° sector, aligning with the dense cellular networking of contemporary 5G systems. Each base station comprises three signal transmitters, each emitting eight SSB beams with minimal time delay. Within each base station, every SSB beam signal is associated with a distinct direction. The three-base station achieves near-complete coverage over the regular hexagonal area.

#### 2.3.1. Coarse Area Localization

To support initial beamforming in 5G NR, base stations employ SS burst sets for SSB transmission and periodically send signals at 20 ms intervals. According to 3GPP TS 38.213 [29] and 5G NR standards, there are five distinct time-domain distribution patterns for SSBs within an SS burst set. Current 5G base stations primarily utilize a high-frequency band scenario, Case C, for SSB transmission, enabling shorter SSB periods and higher beam density. For multi-beam SSB transmission, each SSB is associated with a specific beam direction, enabling the base station to cover different areas by transmitting different beams. This principle enables low-altitude target localization using 5G SSB signals from multiple base stations based on exogenous radar.

The coarse area localization method operates as follows. For the receiving station, under the assumption that interference from multipath clutter and target echoes from other objects within the positioning range is negligible, the signals captured by the receiving station in Figure 2 model include: direct-path signals (SSB5 from gNB1, SSB4 from gNB2, and SSB4 from gNB3), along with reflected target echoes SSB7 from gNB1, SSB2 from gNB2, and SSB4 from gNB3.

Consequently, the receiving station captures a total of six signals. First, we perform operations such as deep learning and cell search, as described in [23], to extract the SSB signals, thereby reducing the cross-correlation search interval and detection time. Next, we apply the ECA based on interference cancellation [23] to detect PCIs of the six SSB beams. Owing to the fixed position of the receiver station, the PCIs of the direct-path SSB beams remain the same. The remaining three beams correspond to the signals that directly face the target. Based on the direction characteristics of the SSB beams, the target’s approximate location can be derived using basic geometric principles.

Signals transmitted from the same base station share the same frequency offset, while those from different base stations exhibit different frequency offsets. Using frequency offset estimation based on joint PSS and SSS, we can find three pairs with relatively similar frequency offsets.

If the target is in motion, such as a UAV, the echo reflected from the target will experience a Doppler shift. As a result, the estimated frequency offset of the target echo signal will be slightly larger than that of the direct-path signal within each frequency-offset pair.

If the target is stationary, the target echo signal travels a greater distance and experiences more signal attenuation due to the geometric principle that the sum of two sides of a triangle exceeds the third side, thus resulting in lower power than the direct-path signal. This distinction allows differentiation between direct-path signals and target echoes in the frequency-offset pairs.

Through the above analysis and operations, the three target echoes can be successfully distinguished from the three direct-path signals. Assuming PCIs of the detected three target echoes are PCI1, PCI2, and PCI3. They correspond to SSB7 from gNB1, SSB2 from gNB2, and SSB4 from gNB3, as shown in Figure 2. Therefore, the approximate location of the target can be determined within the overlapping coverage area formed by the intersection of these three beams. Due to the limited spatial extent of the common coverage area, high positioning accuracy is achieved, facilitating rapid target localization within a prescribed operational range.

#### 2.3.2. High-Precision Localization

To enhance positioning accuracy, we introduce RSRP measurements. In 5G NR, SSB beams are transmitted with specific directivity, distinguishing among them for localization can only provide a coarse estimate of the target’s position, which is insufficient for most outdoor applications. To address this limitation, we construct an RSRP database containing pre-measured RSRP values from multiple reference locations to assist in target localization. This method enables the refinement of the target’s position from the coarse region identified in Section 2.3.1 to a specific point, thereby reducing localization errors and improving overall accuracy.

As shown in Figure 2, we employ a regular hexagonal model formed by three base stations for target localization. The simulation follows Case C configuration, in which each transmitter emits eight distinct SSB beams across a 120° sector, with each beam primarily covering a 15° range.

**Figure 3:**
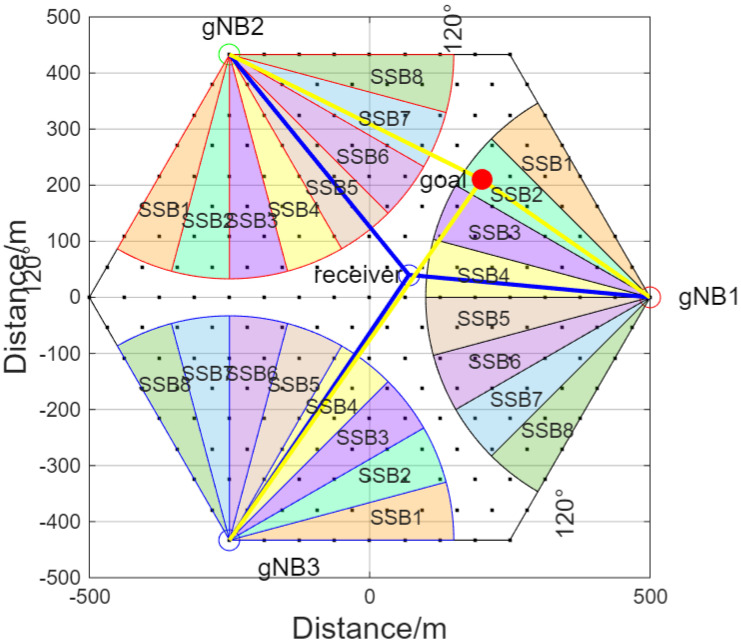
Dense cellular network with 120° sector partitioning.

In order to ensure that the distance between adjacent reference points is small enough so that when the target moves between these points, the RSRP combination of the three-path target echoes exhibits discernible differences, while reducing the time cost to construct the RSRP database, we uniformly select 9 points on each side of the regular hexagon. Based on this, we uniformly place points layer by layer towards the interior, forming a hexagonal lattice of 217 points that serves as the 217 reference points. As shown in Figure 3, the density of reference points not only ensures that each coverage range corresponding to an SSB beam (15° per beam) has reference points, satisfying the beam angle resolution required to ensure the spatial uniqueness of RSRP features, but also significantly reduces time costs associated with excessively dense reference points.

In Figure 3, the red, green, and blue circles represent gNB1, gNB2, and gNB3, located at (500 m, 0 m), (−250 m, 433 m), and (−250 m, −433 m). The receiver station is located at (70 m, 40 m). Assuming the target is at the red point, the blue lines indicate the direct propagation paths, while the yellow lines represent the paths from the base stations to the target.

For each reference point shown in Figure 3, the RSRP values of beams received from different directions and distances are measured and calculated under the hypothetical scenario where the target is located at that point. These average RSRP values are aggregated to construct a database. When a target is located at an arbitrary position in practice, its actually measured RSRP is compared with this database using the normalized Euclidean distance matching method. This allows the target to be located at the closest reference point, thereby enabling effective tracking of low-altitude, slow-moving, and small-sized objects.

We assume that each of the three base stations generates a signal containing only the SSB toward the target, with distinct frequency and timing offsets. In the simulations, we measure RSRP values of target echoes received at the receiving station 25 times for each reference point.

**Figure 4:**
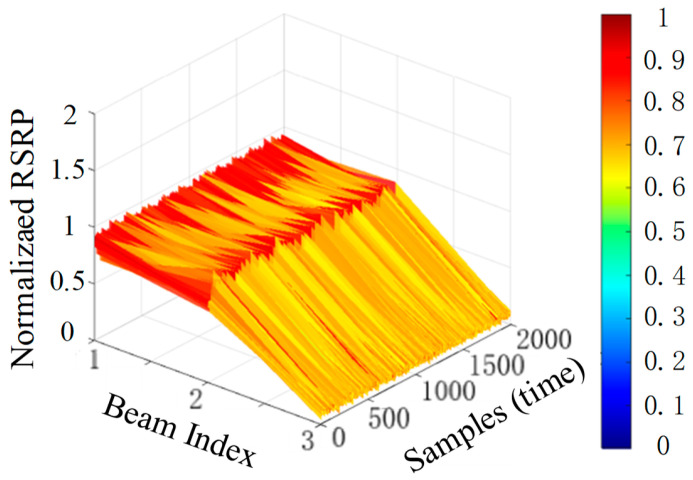
Time-domain normalized RSRP of different signals under 10 dB SNR.

Figure 4 presents simulation results of RSRP calculations for three beams across different time domains at a single location under 10 dB SNR. The results show that the RSRP value exhibits minimal fluctuation over time. Therefore, we adopt the time-averaged RSRP value as the reference RSRP value, which reduces errors and improves robustness.

To mitigate interference from special cases and enhance the robustness of the results, we sequentially measure the RSRP values of the target echoes from the aliased signals. Ignoring multi-path noise effects, the signal received at the receiving station consists of target echoes and three direct-path signals. We selected 217 reference points. For each reference point, we reconstruct the strongest SSB beam in the target echoes, calculate its RSRP value and PCI, then eliminate the interference from already calculated SSB beams, before computing the RSRP value and PCI for the second-strongest SSB beam within the target echo. Since at the same reference point, the PCIs corresponding to the target echo in the received signal remain constant, for the nth reference location, n∈[1,217], the sequence of RSRP values received by the receiving station is:

(29) $$P_{n}=\left[P_{n}^{1},P_{n}^{2},P_{n}^{3}\right]$$

where $P_{n}$ is the average RSRP matrix of the target echoes received by the receiving station when the target is at the nth reference point, and $P_{n}^{h}$ is the average RSRP value of the target echo signal with the hth largest PCI, h∈{1,2,3}.

Then, the RSRP sequence $P_{ref}$ corresponding to all reference positions is:

(30) $$P_{ref}=\left[P_{1};P_{2};P_{3};\ldots ;P_{217}\right]\in {\mathbb{R}}^{217\times 3}$$

Similarly, we select M test positions. The RSRP sequence $p_{M}$ received by the receiving station with the target at the Mth reference position can be calculated as:

(31) $$p_{M}=[p_{M}^{1},p_{M}^{2},p_{M}^{3}]$$

where $p_{M}$ is the RSRP matrix of the target echoes received by the receiving station when the target is at the Mth test position, $p_{M}^{h}$ is the RSRP value of the target echo signal with the hth largest PCI, h∈{1,2,3}. The RSRP sequence $p_{obj}$ corresponding to all test positions is:

(32) $$p_{obj}=[p_{1};p_{2};\ldots ;p_{M}]\in {\mathbb{R}}^{M\times 3}$$

Based on the coarse localization of Section 2.3.1, we determine the general range of the target’s location and find the reference points within this range. Then we extract the corresponding RSRP values at these reference points from Equation (29). Then their corresponding RSRP matrix $P^{\prime }$ can be described as:

(33) $$P\prime =\left[\begin{array}{ccc} P_{o_{1}}^{1} & P_{o_{1}}^{2} & P_{o_{1}}^{3}\\ \ldots & \ldots & \ldots \\ P_{o_{m}}^{1} & P_{o_{m}}^{2} & P_{o_{m}}^{3} \end{array}\right]$$

Assuming the range from Section 2.3.1 contains m reference points $\left(o_{1}\sim o_{m}\right)$, $P_{o_{m}}^{n}$ denotes the RSRP value corresponding to the signal with the nth largest PCI at the position $o_{m}$.

We calculate the standard Euclidean distance between $p_{obj}$ and $P^{\prime }$. The reference point with the smallest distance is identified as the estimated location of the target, which is relatively more precise.

In practical scenarios, rapid random jitter occurs during signal transmission due to factors such as channel interference, noise, and hardware. This instability in the received signals, in turn, affects the value of the key parameter RSRP. We consider small-scale linear proportional jitter for simulation. y(n) is the initial signal before jitter, $y_{jitter}(n)$ is the signal after jitter, $\alpha_{dB}$ is the magnitude of jitter in dB, and X∼N(0,1) is a random variable from the standard normal distribution. Then the amplitude jitter factor $\alpha_{jitter}$ can be expressed as:

(34) $$\alpha_{jitter}=10^{\frac{\alpha_{dB}}{20}\cdot X}$$

The signal $y_{jitter}(n)$ after amplitude jitter is:

(35) $$y_{jitter}(n)=y(n)\cdot \alpha_{jitter}$$

Subsequent processing is then applied to $y_{jitter}(n)$ in the same way.

## 3. Results

### 3.1. Performance of Frequency Offset Estimation Under Different Subcarrier Spacings

**Figure 5:**
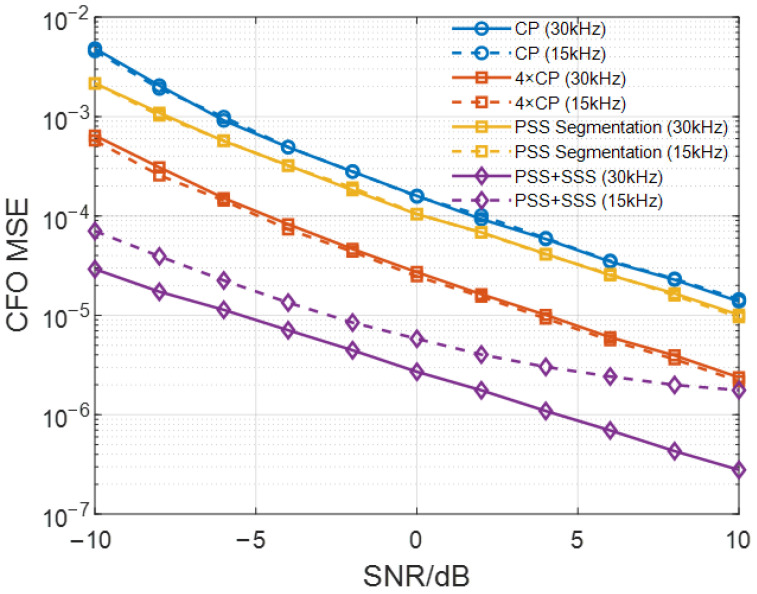
CFO estimation performance comparison under different subcarrier spacings in an AWGN channel.

We compared the frequency offset estimation performance based on a single cyclic prefix (CP), four CPs, PSS sequence segmentation, and joint PSS and SSS sequences in an additive white Gaussian noise (AWGN) channel under 30 kHz and 15 kHz subcarrier spacings, which is shown in Figure 5. We set the number of Monte Carlo simulations to 3000, the normalized frequency offset to 0.02, and used the mean squared error (MSE) as the performance evaluation metric.

In Figure 5, dashed lines and solid lines represent the MSE under 15 kHz and 30 kHz subcarrier spacings, respectively. As expected, the frequency offset estimation accuracy of all methods improves with increasing SNR. Due to the limited length of the CP and the PSS sequence, the signal available for estimation is insufficient, resulting in relatively low estimation accuracy for these two methods. Non-coherent accumulation over four CPs provides a modest enhancement. Notably, the method utilizing the joint PSS and SSS sequences achieves the highest accuracy. As a result, we employ this joint method to estimate the frequency offset in our further simulation.

It is also noteworthy that the estimation accuracy of the CP-based and PSS segmentation-based methods remains almost the same under both subcarrier spacings. In contrast, the joint PSS and SSS-based method exhibits slightly better performance at 30 kHz than at 15 kHz.

### 3.2. Performance of Multi-Beam Extraction

To validate the performance and feasibility of the multi-base station and aliased signals model, we simulated the aliased signals received from three co-channel cells at the receiving station under varying SNR conditions. We set the number of Monte Carlo simulations to 3000 and defined the performance metric as the probability of correct detection for each cell. Successful detection of a cell was defined by two conditions: the detected PCI matches the cell’s theoretical PCI value, and the coarse synchronization point identified during the PSS search lies within the theoretical cyclic prefix range.

**Figure 6:**
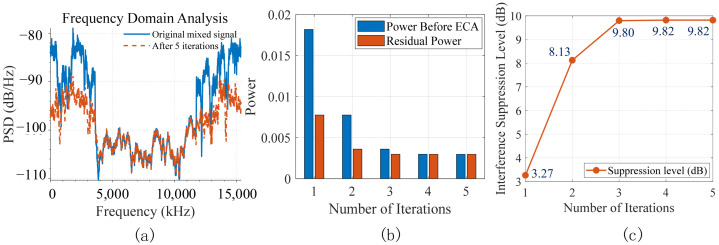
The performance of ECA: (**a**) PSD of the aliasing signal before and after the ECA; (**b**) power of the initial signal before each iteration and the residual signal after each iteration; (**c**) the interference suppression level of each iteration.

Assuming the three co-channel cells have PCIs of 0, 5, and 10, the specific simulation parameters for these cells are detailed in Table 1 below:

To evaluate the performance of ECA, we set the number of iterations to 5 and calculated the power spectral density (PSD) before and after the ECA, along with the signal power and residual power at each iteration. The results are presented in Figure 6a,b. The interference suppression level relative to the original signal for each iteration is also presented in Figure 6c. For the aliased signal, when the number of iterations reaches 4, all cells have been detected. The CRC check fails, and the signal remains essentially unchanged between iterations. Therefore, the optimal number of iterations is 3.

**Figure 7:**
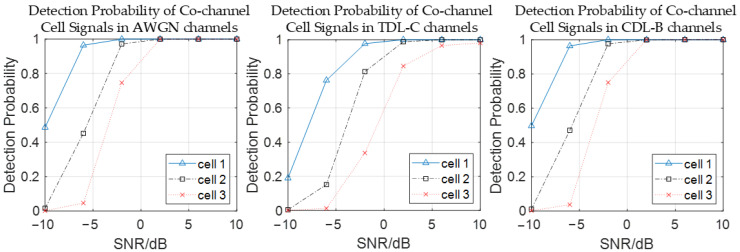
Detection probability of co-channel cell signals in different channels.

Additionally, Figure 7 shows the detection probabilities of three co-channel cell signals in AWGN, tapped delay line model C (TDL-C), and clustered delay line model B (CDL-B) channels. Observations reveal that the detection probability for each cell is highest under the AWGN channel and lowest under the TDLC channel at the same SNR, due to multipath fading and Doppler effects. Nevertheless, when the SNR exceeds 2 dB, the detection probability for each cell still approaches 100%, demonstrating the high accuracy of the ECA in signal reconstruction and interference cancellation even under fading and Doppler conditions.

**Figure 8:**
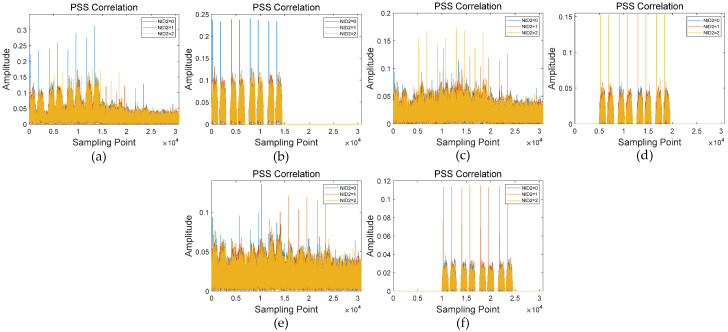
Examples of cross-correlation between the signal after each stage of reconstruction and cancellation: (**a**) initial signal; (**b**) first-stage signal reconstruction; (**c**) signal after one-stage reconstruction and cancellation; (**d**) second-stage signal reconstruction; (**e**) signal after two-stage reconstruction and cancellation; (**f**) third-stage signal reconstruction.

To better visualize the interference cancellation performance of the ECA, the simulation results for detecting the three cells under a 20 dB SNR in a TDL-C channel are presented as follows. Firstly, we cross-correlated the aliased signal with three candidate PSS sequences, as shown in Figure 8a. The blue, red, and yellow lines represent cross-correlation results with candidate PSS sequences of NID2 = 0, 1, and 2. Next, we calculated its PCI and detected other information of the strongest SSB signal. Using the ECA, we reconstructed the signal with the highest power, cell 1 in Table 1 (PCI = 0, NID2 = 0, carrier frequency offset = 15 kHz, timing offset = 0), as shown in Figure 8b, and eliminated this signal from the aliased signal. Then we did the same operations on the second-strongest signal iteratively until all the signals had been detected. To verify the complete elimination of this strongest signal, we cross-correlated the remaining signal with the three candidate PSS sequences, as shown in Figure 8c. We could see that after one reconstruction and cancellation step, the strongest cell signal with no timing offset in Figure 8b was substantially eliminated in the signal of Figure 8c. Similarly, in Figure 8d–f, we sequentially reconstructed and eliminated signals from the second and third-strongest cells. As shown in Figure 8, this method effectively detects all overlapping cell signals, demonstrating its feasibility.

We further evaluated the performance of the ECA using aliased signals from six co-channel cell signals. The basic parameters of the signals are displayed in Table 2.

**Figure 9:**
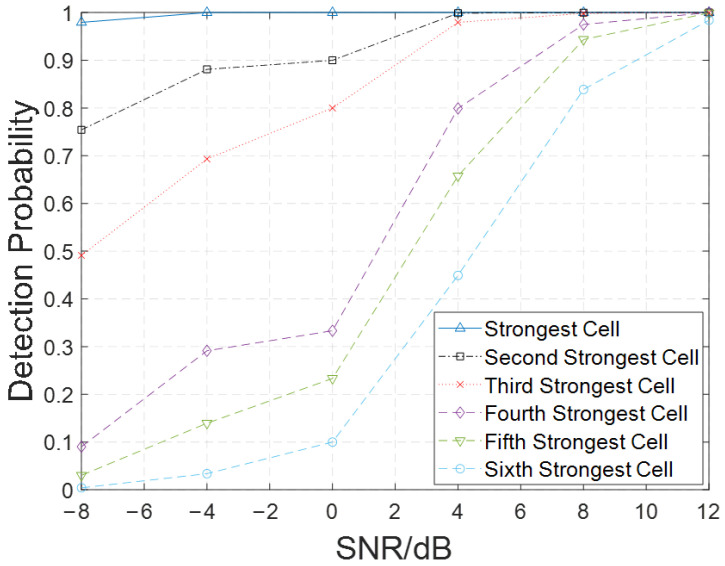
Detection probability of six co-channel cell signals.

Figure 9 shows the detection probability of cell signals. We find that cells with strong signals maintain a high detection rate even at low SNR levels; cells with weak signals require a higher SNR to achieve the same detection probability. After ECA iterations, the detection probability of the cells increases as the SNR improves.

These findings demonstrate that the proposed method effectively extracts comprehensive SSB-related information from the received aliased signal, highlighting the receiver’s strong capability for information acquisition in aliased signals. Simulation results indicate that the three PCIs corresponding to the SSBs of the target echoes in the received signal can be used to determine the intersection range of the fan-shaped sections, thereby pinpointing the coarse location of the target. This method exhibits both scientific validity and practical feasibility.

### 3.3. RSRP Calculation

With the position of the receiving station fixed, the direct-path signals remain consistent across different time instances. Therefore, we eliminate the direct-path signals in the received signal for analysis and focus solely on the three SSB signals within the target echoes. We strictly select parameters based on realistic 5G base-station configurations and empirical settings. In line with the 3GPP technical specifications, we select Frequency Range 1 (FR1), also called “Sub-6GHz band”, with a signal bandwidth of 100 MHz and a sampling frequency of 122.88 MHz. All the specific parameters used are detailed below in Table 3.

**Figure 10:**
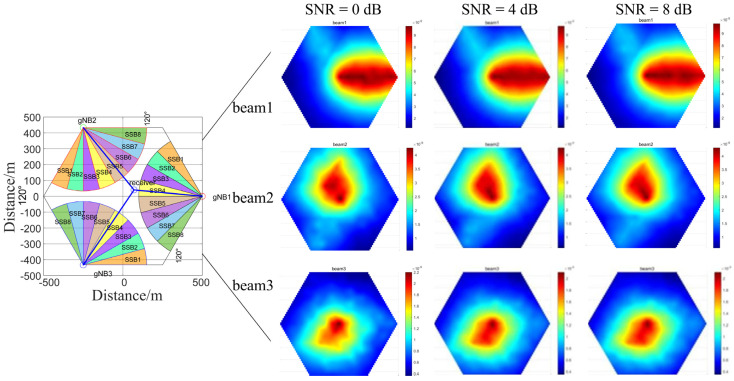
Spatial distribution of RSRP under varied SNR conditions in AWGN channel.

Figure 10 shows the spatial distribution of RSRP at different SNRs in an AWGN channel. Beam 1 represents the signal transmitted from gNB1 and received after reflection from the target, whereas beam 2 and beam 3 represent signals from gNB2 and gNB3, respectively, undergoing the same reflection mechanism. Since RSRP values correlate with propagation distance, regions with higher RSRP values in beam1 plots of Figure 10 are situated closer to gNB1, which is in accordance with theoretical predictions.

Let h represents the total propagation path length from the base station to the target and then to the receiving station. The RSRP value increases as h decreases. The observable relationship between RSRP and position demonstrates the feasibility of using RSRP values for target localization.

### 3.4. Simulations

The following presents a simulation for precise positioning based on RSRP values. Initially, we did not consider amplitude jitter during signal transmission. Since the 5G base stations are stationary and positioned at a fixed distance from the receiving station, the RSRP of the direct-path signals remains essentially constant. The simulation was therefore conducted after removing the direct-path signals from the received signal.

We simulated the aliased target echo signals originating from three base stations. For each of the 217 reference points, the target position was estimated using a model that accounts for signal propagation through an AWGN channel, receiver thermal noise, and transmission attenuation.

**Figure 11:**
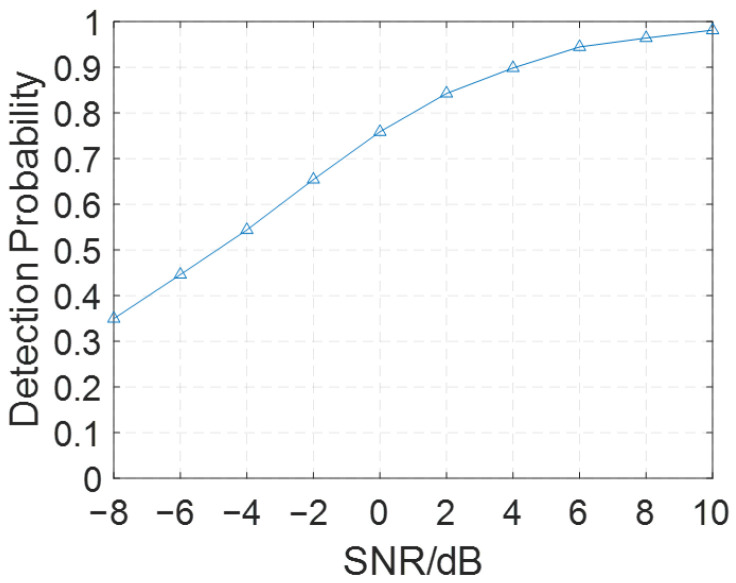
Localization accuracy at 217 reference positions under varied SNR in the AWGN channel.

Figure 11 illustrates the probability that the estimated position, obtained by minimizing the Euclidean distance between the average RSRP value and the measurement results, coincides with the true target position.

As shown in Figure 11, the proposed method achieves high accuracy by utilizing the RSRP values. When the SNR exceeds 4 dB, the localization accuracy rate exceeds 90%. Furthermore, higher SNR values are associated with increased accuracy in pinpointing the true target position.

**Figure 12:**
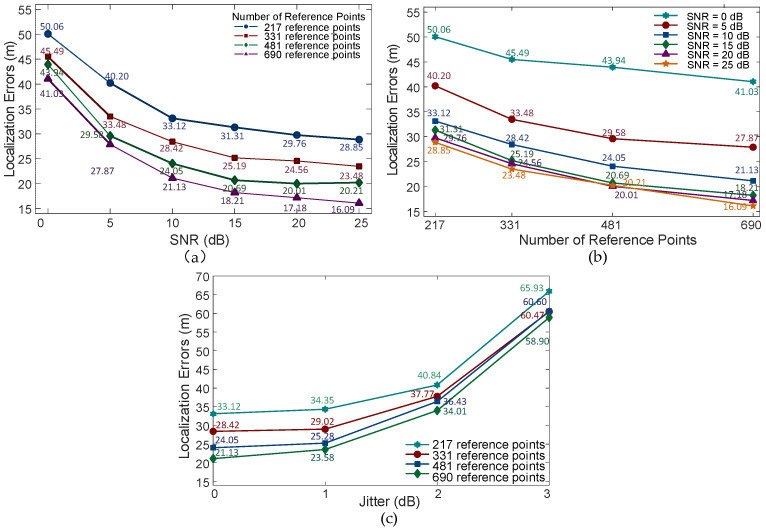
Localization errors under different conditions. (**a**) Localization errors under different SNR levels; (**b**) Localization errors under different numbers of reference point; (**c**) Localization errors under varying degrees of signal jitter.

We performed simulations by uniformly and randomly selecting 4961 points within the hexagonal positioning area as true target locations. The positioning performance was evaluated using the mean error between the true and estimated positions. We uniformly selected 217, 331, 481, and 690 reference points within the area. The results are illustrated in Figure 12.

Figure 12a,b shows the localization errors under different SNR conditions for varying numbers of reference points. Figure 12c shows localization errors at 10 dB SNR with varying degrees of signal jitter and different numbers of reference points.

As shown in Figure 12a, the localization errors decrease as the number of reference points increases, which is consistent with theoretical predictions. Meanwhile, in Figure 12b, the localization errors exhibit a consistent decrease with rising SNR for varying numbers of reference points.

Simultaneously, due to signal jitter during propagation, the RSRP values at different locations are affected. We investigated signals with varying amplitude jitters at 10 dB SNR. Figure 12c illustrates that localization errors remain low when signal jitter ranges between 0 dB and 1 dB, demonstrating the effectiveness of the proposed method in mitigating aliasing effects.

### 3.5. Error Analysis

**Figure 13:**
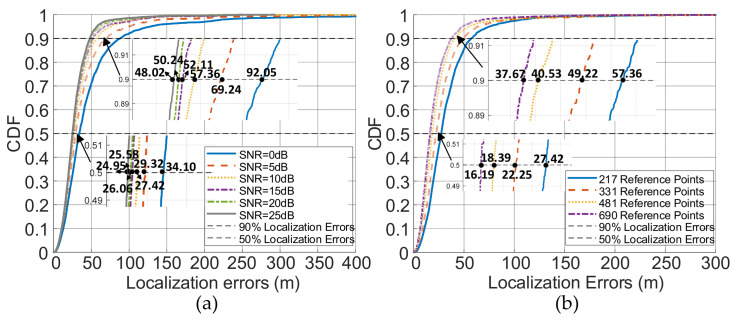
Cumulative Distribution Function (CDF) comparisons of the localization errors under different conditions: (**a**) Comparisons of the localization errors under different SNR conditions when adopting 217 reference points using the CDF; (**b**) Comparisons of the localization errors for different numbers of reference points at 10 dB SNR.

To better quantify how SNR, the number of reference points, and jitter magnitude influence localization errors, we conduct an error analysis. As illustrated in Figure 13, the 50% and 90% localization errors under different SNR conditions corresponding to 217 reference points range from 24.95 m to 34.10 m and 48.02 m to 92.05 m, respectively. In contrast, when varying the number of reference points from 217 to 690 at 10 dB SNR, the corresponding errors range from 16.19 m to 27.42 m and from 37.67 m to 57.36 m.

**Figure 14:**
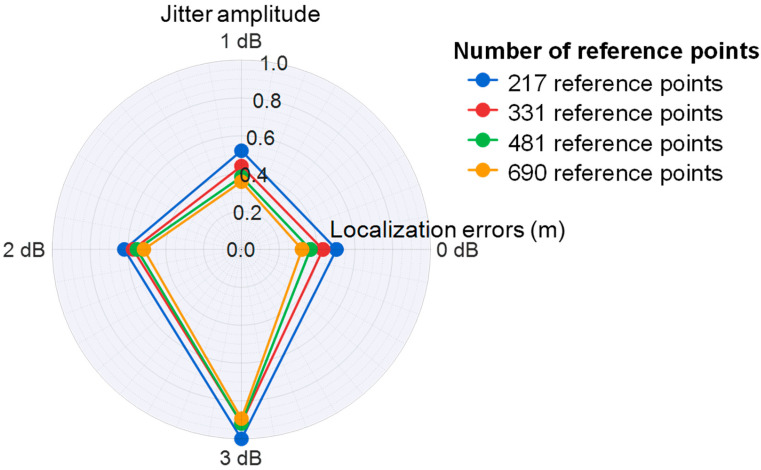
Localization errors with varying degrees of signal jitter at 10 dB SNR.

To better investigate the impact of the signal jitter amplitude on localization errors, we plotted a radar chart. Figure 14 demonstrates localization errors with varying degrees of signal jitter at 10 dB SNR. The axes represent the amplitude of signal jitter, while the radial scale indicates the normalized localization errors.

**Figure 15:**
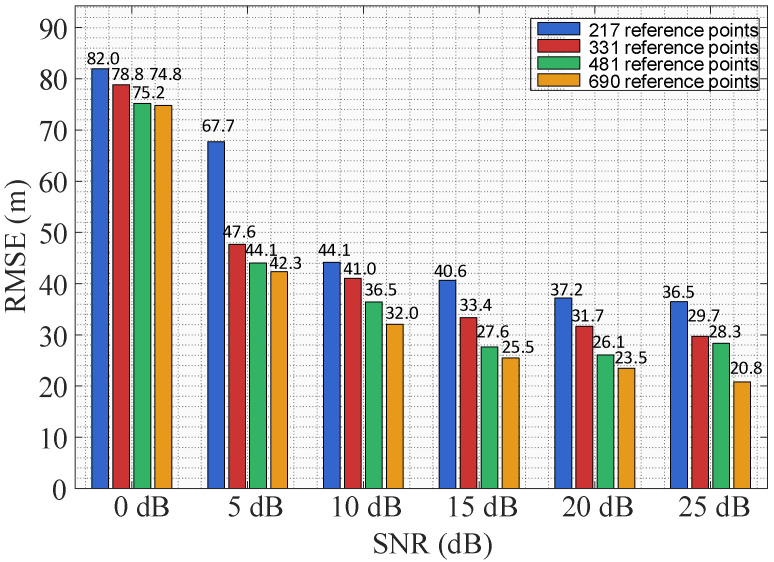
RMSE comparison under different conditions.

Based on the localization method proposed in this paper, Figure 15 compares the root mean square error (RMSE) of localization errors under different numbers of reference points and varying SNR conditions. As is seen from the results, the localization performance improves almost monotonically with increasing SNR and a larger number of reference points.

## 4. Discussion

### 4.1. Comparison of Positioning Results Using Different RSRP-Matching Methods

In Section 3.3, we randomly and uniformly selected 4961 points within the area as the test positions. We then identified the best-matching reference point in the database based on the measured RSRP and assigned it as the target’s position. The localization errors obtained from simulations varied depending on the employed identification method. We primarily discussed Euclidean distance, normalized Euclidean distance, and the K-means iteration method. Table 4 shows the localization errors and anti-aliasing performance measured using different methods for 217 reference points under varying signal jitters at 10 dB SNR.

Table 4 demonstrates that the normalized Euclidean distance method achieves the best localization performance during signal jitter, showing significant potential in high-signal-jitter scenarios. The localization errors of the normalized Euclidean distance method are significantly lower than those of the Euclidean distance method, which indicates that normalization effectively mitigates the impact of jitter and enhances anti-aliasing capability while maintaining high computational efficiency. The Euclidean distance method exhibits poor performance, with errors increasing sharply under severe signal jitter conditions and causing larger localization errors than both the normalized Euclidean distance method and the K-means iteration method.

### 4.2. Computational Complexity and Runtime Analysis

To evaluate the complexity of the method proposed in this paper, all the simulations are run on an Intel(R) Core (TM) i5-1340P @ 1.9 GHz CPU.

#### 4.2.1. RSRP Dataset Construction

Before locating the target, we need to calculate the RSRP values in the case of n reference points. The total dataset construction process is divided into two parts: PSS/SSS detection and ECA Interference cancellation. Table 5 demonstrates the computational complexity of the RSRP dataset construction process.

#### 4.2.2. RSRP Matching

We evaluated the computational complexity and runtime of Euclidean distance, normalized Euclidean distance, and K-means iteration methods used for point matching, as discussed in Section 2.3.2.

Table 6 compares the computational flexibility of using the three RSRP-matching methods. Specifically, the computational complexity of the Euclidean distance method is denoted as $O\left(M\times n\right)$, and for the normalized Euclidean distance method, it is denoted as $O\left(M\times n+M+n\right)\approx O\left(M\times n\right)$. However, the computational complexity of the K-means iteration method is $O\left(I\times M\times n\right)$, where I denotes the measured average convergence iterations.

In our simulation, we set M to 4961 and n to 217. We conduct 100 times point-matching operations at 0 dB SNR. The empirical simulation runtime matches the theoretical computational complexity, as displayed in Table 6. The runtimes of Euclidean distance and normalized Euclidean distance are almost the same, demonstrating that the runtime of global normalization is negligible. However, the runtime of the K-means iteration method is much greater than the other two.

#### 4.2.3. Average Processing Delay

**Figure 16:**
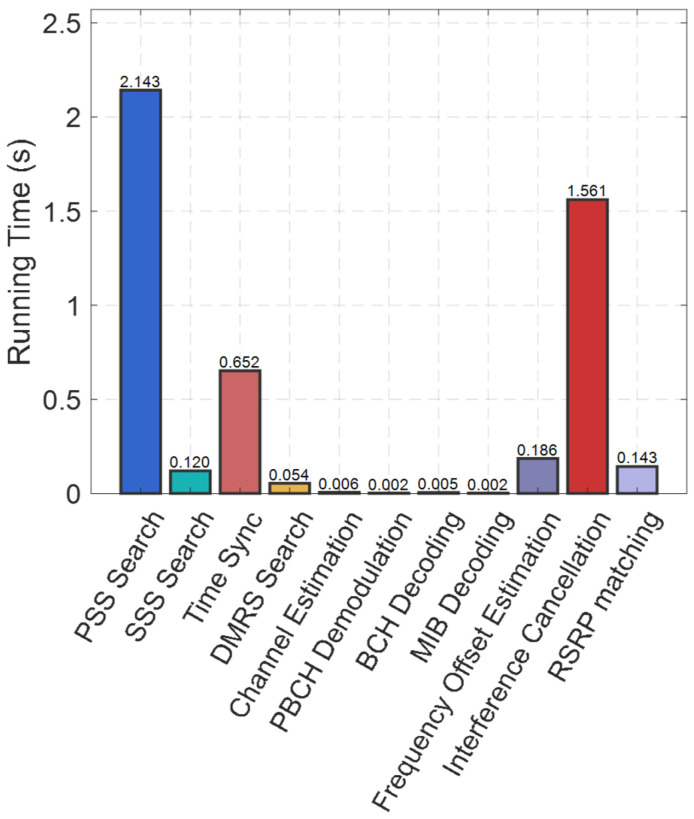
Average running time of each procedure.

The signal processing can be divided into several parts: PSS/SSS detection, ECA interference cancellation (including time synchronization, DM-RS search, channel estimation, PBCH demodulation, BCH decoding, MIB decoding, frequency offset estimation, and interference cancellation), and RSRP matching using the normalized Euclidean distance method. Figure 16 shows the average running time of each procedure.

Based on the three-base station model, we conduct 1000 simulations, with three signals being reconstructed and cancelled per simulation. Each signal costs 4.73 s, resulting in an average delay of 14.19 s. The RSRP matching costs 0.14 s. To further reduce processing time, after the coarse time synchronization through PSS search, we perform frequency-domain complex correlation for the SSS search. From Figure 16, we can see that PSS search and interference cancellation account for a significant portion of the total time, accounting for 78.3% of the total duration. The running time can be further reduced by down-sampling.

### 4.3. Comparison of Positioning Errors Among Different Methods

This study compares the positioning performance of the proposed algorithm with commonly used 5G positioning methods: the Chan algorithm based on TDOA, the Fang algorithm based on TDOA, and the standard OTDOA algorithm.

**Figure 17:**
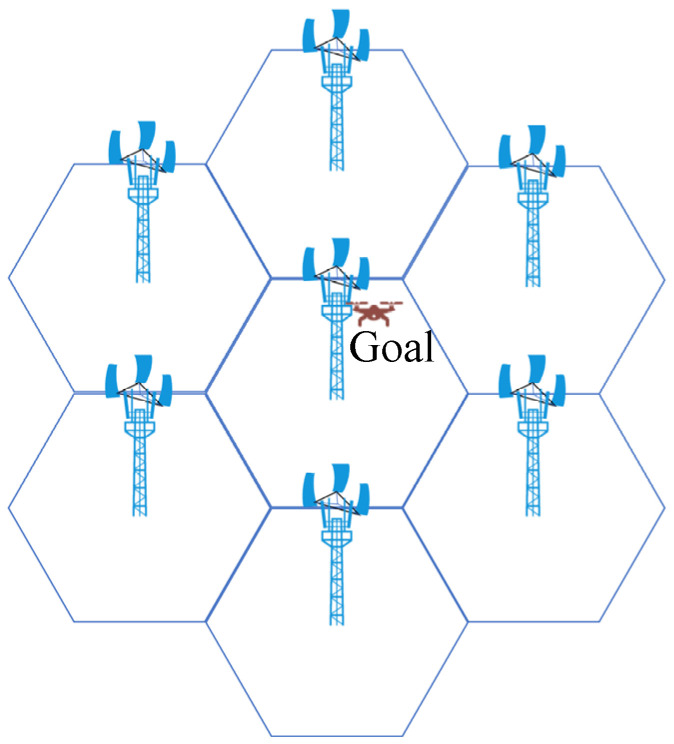
7-Base Station Model with Signal Aliasing in 5G Positioning.

To accurately simulate cellular network structures, we employed a seven-base-station model for calculating the TDOA-based Chan and Fang algorithms, OTDOA, and the proposed algorithm, as shown in Figure 17. The positions of the seven base stations are (0, 0), (433 m, 250 m), (0, 500 m), (−433 m, 250 m), (−433 m, −250 m), (0, −500 m), and (433 m, −250 m).

**Figure 18:**
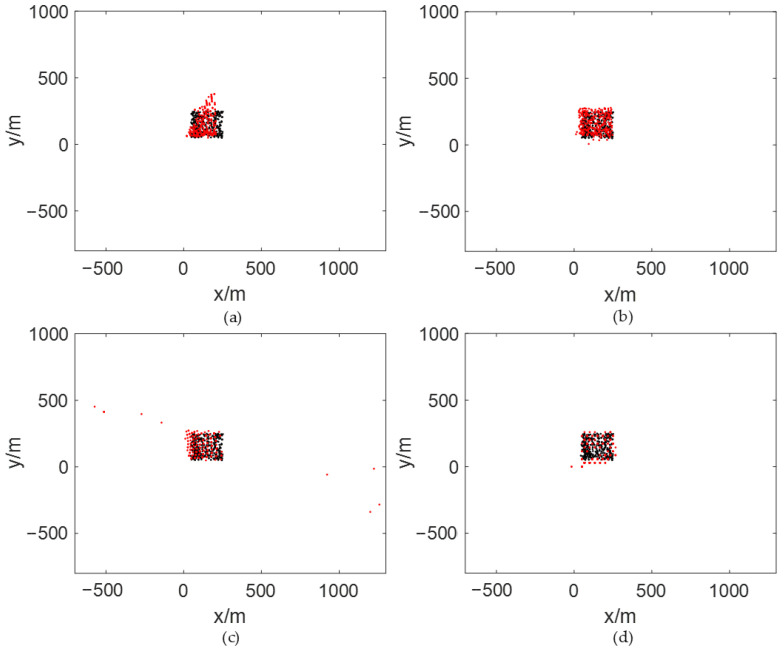
Localization results. (**a**) Localization results using TDOA-based Chan algorithm; (**b**) Localization results using TDOA-based Fang algorithm; (**c**) Localization results using OTDOA algorithm; (**d**) Localization results using the method proposed in this paper.

Assuming a simulated SNR of 20 dB, the black “⋅” marks in Figure 18a–d represent the selected target positioning points. The red “⋅” marks denote the estimated positions obtained using the TDOA-based Chan algorithm, TDOA-based Fang algorithm, OTDOA algorithm, and the method proposed in this paper, respectively.

Table 7 presents the RMSE values and average positioning errors obtained through simulation for different localization methods under 20 dB SNR. Based on the physical limitations and the expected performance of the system, the intersite distance is 500 m in our model displayed in Figure 17. Any localization result with an error greater than 1000 m is physically implausible, so we remove outliers with localization errors exceeding 1000 m.

Comparing the simulation results, we find that the Chan-TDOA and OTDOA algorithms produce some outliers in their positioning results, where the estimated location points deviate significantly from the actual positions of the target. Among these, the OTDOA algorithm exhibits the highest number of outliers, accounting for 4.7%, while the Chan-TDOA has an outlier rate of 1.0%.

Further comparison shows that the localization errors obtained by the proposed method are smaller than those of the Fang-TDOA, Chan-TDOA, and the OTDOA algorithm, yielding more accurate localization results. Compared with the Fang algorithm, the localization error is reduced by 18.2%; compared with the OTDOA method, it is reduced by 20.5%. As shown in Figure 18c, OTDOA-based target localization is prone to large outliers, leading to inaccurate results. In contrast, the proposed method achieves robust performance by precisely locating the target to the reference point, minimizing the occurrence of outliers. Although the localization errors of the proposed method are slightly higher than those of the Chan algorithm, the proposed method exhibits lower RMSE values, indicating that it is less prone to large errors. Furthermore, the method proposed in our work does not require the target to emit or receive electromagnetic waves, which broadens its application scope and avoids additional electromagnetic pollution, making it highly significant.

### 4.4. Potential Challenges and Possible Improvements

Although we have proposed a multi-base station localization method based on exogenous radar, several issues and challenges remain. The primary limitation is the multipath effect. When multipath occurs, the multipath clutter received by the receiving station overlaps with the target echo, making them potentially indistinguishable. Furthermore, when more SSB signals overlap or when there is a significant power disparity between signals, the ECA cannot fully separate different signals, leading to increased localization errors. What’s more, an appropriate number of reference points must be selected to avoid unnecessary resource expenditure. This study is currently based only on simulations and lacks experimental measurements in real-world environments. To address these issues and challenges, future research will focus on upgrading the ECA to detect more signals, integrating the multi-base-station model with machine learning to enhance robustness and reduce the impact of multipath effects on results, conducting hardware experiments in practical environments, and taking hardware heterogeneity and inconsistent signal parameters in real 5G networks into consideration.

## 5. Conclusions

This study analyzes the technology and methods for multi-base station target positioning using the SSBs of 5G signals. By leveraging existing 5G base stations, the method introduces an SS burst set transmitted via SSB. Drawing on the ECA, it addresses the challenge of identifying overlapping SSB signals. Furthermore, by identifying base stations that transmit different SSB signals toward distinct directions, the method determines the coarse location of the target. To improve positioning accuracy, we employ the RSRP of SSB beams as an auxiliary locator. By analyzing variations in RSRP magnitudes from multiple received signals, we significantly narrow the positioning range, enabling high-precision target localization. This approach utilizes existing signals without generating additional electromagnetic radiation, thereby ensuring environmental sustainability. In summary, this research offers substantial advantages and holds great significance for low-altitude target detection.

## Figures and Tables

**Table 1:** Simulation parameters of three co-channel cell signals.

Simulation Parameter Name	Cell 1	Cell 2	Cell 3
Physical Cell ID	0	5	10
Relative Power (dB)	0	−4	−6
Carrier Frequency Offset (kHz)	15	7.5	0
Timing Offset (ms)	0	0.65	1.3
Sampling Rate (MHz)	15.36		
Subcarrier Spacing (kHz)	30		
SSB Transmission Mode	8-beam Case C		
SSB Periodicity (ms)	20		
Transmission Channel Type	AWGN, TDL-C, CDL-B		

**Table 2:** Simulation parameters of six cells on the same frequency.

Simulation Parameter Name	Cell 1	Cell 2	Cell 3	Cell 4	Cell 5	Cell 6
Physical Cell ID	0	5	10	15	20	25
Power (dBm)	13	12	11	8	7	6
Carrier Frequency Offset (kHz)	0	1	2	3	4	5
Timing Offset (ms)	0	0.65	1.3	1.95	2.6	3.25
Sampling Rate (MHz)	15.36					
Signal Bandwidth (MHz)	10					
Subcarrier Spacing (kHz)	30					
SSB Transmission Mode	8-beam Case C					
SSB Periodicity (ms)	20					

**Table 3:** Simulation parameters for three-base-station signal scenarios.

Simulation Parameter Name	gNB1	gNB2	gNB3
Frequency Offset (kHz)	0	3	5
Time Offset (us)	0	65	130
Power (dBm)	38	35	32
Sampling Rate (MHz)	122.88		
Signal Bandwidth (MHz)	100		
Subcarrier Spacing (kHz)	30		
SSB Periodicity (ms)	20		
SSB Pattern	Case C		
Number of Transmitted SSBs	8		
FFT Size	4096		
Cyclic Prefix	Normal		

**Table 4:** Performance comparison of anti-aliasing methods for signal localization under 10 dB SNR.

	Jitter (dB)	0 dB	1 dB	2 dB	3 dB
Method					
Euclidean distance		44.0 m	44.5 m	52.7 m	74.9 m
Normalized Euclidean distance		33.1 m	34.3 m	40.8 m	65.9 m
K-means iteration		40.8 m	41.7 m	49.0 m	70.4 m

**Table 5:** The computational complexity of the RSRP dataset construction process.

Process	Computational Complexity
PSS detection	$O\left(n\times F_{s}\times T_{p}\times N_{s}\times N_{PSS}\right)$
SSS detection	$O\left(n\times F_{s}\times T_{p}\times N_{p}\times N_{SSS}\right)$
Interference cancellation	$O\left(n\times L_{\max}\times F_{s}\times T_{p}\times {\left(2M_{t}\right)}^{2}\right)$

where $F_{s}$ is the sample rate (equal to 122.88 MHz); $T_{p}$ is the transmission period of an SS burst set (equal to 20 ms); $N_{PSS}$ is the number of candidate PSS sequences (equal to 3); $N_{SSS}$ is the number of candidate SSS sequences (equal to 336); $M_{t}$ is the number of delay taps used for interference cancellation.

**Table 6:** Comparison of time complexity and runtime among the three methods.

Method	Time Complexity	Runtime/s
Euclidean distance	$O\left(M\times n\right)$	0.0142±0.0024
Normalized Euclidean distance	$O\left(M\times n+M+n\right)$	0.0143±0.0027
K-means iteration	$O\left(I\times M\times n\right)$	0.3983±0.0336

**Table 7:** RMSE values and average positioning error simulated for different positioning methods at 20 dB SNR.

Positioning Method	RMSE (m)	Localization Errors (m)
Chan-TDOA Algorithm	60.5 m	33.1 m
Fang-TDOA Algorithm	74.1 m	50.1 m
Observed Time Difference of Arrival (OTDOA)	115.8 m	51.6 m
Algorithm Studied in This Paper	50.8 m	41.0 m

## Data Availability

The data that support the findings of the study are available in Mendeley Data at https://doi.org/10.17632/62zw9sw935.1.

## Abbreviations

The following abbreviations are used in this manuscript:

SSB	Synchronization Signal Block
TDOA	Time Difference of Arrival
OTDOA	Observed Time Difference of Arrival
RSRP	Reference Signal Receiving Power
RSRQ	Reference Signal Reception Quality
PSS	Primary Synchronization Signal
SSS	Secondary Synchronization Signal
PBCH	Physical Broadcast Channel
DM-RS	Demodulation Reference Signal
ECA	Extensive Cancellation Algorithm
MIB	Master Information Block
